# Traité de Nosographie Médicale

**Published:** 1846-07

**Authors:** 


### Traité de Nosographie Medicale. Par *J. Bouillaud.* Tomes I. II. III. IV. V. Paris et Londres, 1846. Baillière.

This work possesses no ordinary pretensions to the notice of the professional critic. The mere circumstance of its consisting of five bulky volumes, which profess to treat of the entire subject of medical Nosography, is in itself sufficient to attract his attention. It is moreover the production of a physician, who occupies a prominent position in the

French metropolis; for M. Bouillaud is not only a Professor to the Faculty, and attached to one of the largest hospitals in that city, not to mention his being a member of the Chamber of Deputies, but he is also well known as a most active and busy author. His previous works have gained for him the reputation of being a quick observer, a bold unhesitating practitioner, and a rapid and not inelegant writer. Perhaps no man in the medical world of Paris has had, during the last 20 years, his name more frequently and more prominently before the public eye. Besides numerous contributions to various journals—he was the editor, if we are not mistaken, of one of them—he has published distinct treatises on Fever, on Rheumatism, and on Diseases of the Heart; an ample work on Clinical Medicine, and an elaborate disquisition, in two large volumes, on Medical Philosophy. The present work embodies the sum and substance of all his previous writings, and contains besides, as a matter of course, much new matter on other subjects which he had not previously discussed in writing. It professes, as we have said, to be a complete systematic exposition of the present condition of medical science, and evidently aims at taking the place of the *Nosographie Philosophique* of the celebrated Pinel—a book, it is well known, of very high merit and extensive circulation; for it passed through no fewer than six editions, and, for nearly a quarter of a century, was the leading authority on the theory and practice of physic in all the medical schools of France.

One of the first impressions on the mind of the reader, from the perusal of the present work, is, that it is much too controversial to be generally useful, or to warrant the expectation of its ever becoming a standard treatise. It is written too much for the present day, and for a particular class or school; it is the exponent rather of one section, than of the entire body, of the medical profession of its country. M. Bouillaud's acquaintance with the literature of medicine is moreover far too scanty to have justified any reasonable hopes of his success in reviewing the entire subject of nosography. He seems to know little or nothing of what has been going on in other countries save his own, during the course of the present century. Whether this ignorance arises from his time having been completely absorbed in the duties of active practice, or from a patriotic disregard of all foreign literature, we cannot pretend to say. The defect is certainly an unfortunate one for the author himself, if he has ever hoped to be known beyond the limits of "la belle France;" and that his publisher, at least, fondly entertains this laudable hope is pretty obvious from the somewhat curious advertisement on the fly-leaf of the first volume—that this Treatise "se trouve" not only in all the leading cities of the continent of Europe, an enumeration of which is given along with the names of the resident booksellers, but also in Boston and New York, in Mexico and Valparaiso, in the New World. We trust that the spirited bibliopole's expectations may be realised; but we must not forget to add, at the same time, that, just in proportion to the expected wide circulation of any didactic work, so much the more necessary it is for the medical critic to scrutinise its contents, and to estimate, with candour yet impartiality, the claims which it puts forth to public approbation. We have said that the present work is of a much more controversial character than well suits a systematic treatise. Scores and scores again of pages are taken up with a review of the writings of Bichat, Pinel, Broussais, and one or two other writers; and then there is such an unmerciful repetition, on many occasions, upon the same subject, that the reader often feels indignant at his patience being so unnecessarily taxed.

Another prominent feature of this Nosography is the ever-renewed and almost ceaseless attempt to reduce all diseases to a few very simple types; whose existence, it is alleged, may very generally be discovered by the physician versed in all the exact knowledge of modern times, and the discovery of which is supposed to lead with an almost inevitable precision to a course of successful treatment. The author's grand aim and object are, as he often tells us, to establish *unity* and a sort of mathematical precision and exactitude in medical practice. Diseases are, in his view, so many entities, that have very generally certain steady characters, and that may be made subject to certain curative and therapeutic rules, which it is the object of the philosophic physician to discover. We shall find, as we proceed, that he talks of an almost unerring certainty in the cure of some maladies, by following a particular line of treatment. He goes further than Bichat, who anticipated that the time might come when the diagnosis of diseases would be so rigorously accurate as to entitle medicine, in one respect at least, to take its place among the exact sciences. M. Bouillaud would extend this remark to the department of therapeutics. He seems, indeed, to regard the human body as an engineer regards any piece of finely-adjusted machinery, which is every now and then subject to casualties and injuries; but these may generally be rectified by the skill of the wise and cunning workman. No allowance is ever made for the influence of the mind and feelings on the corporeal tenement, or of those subtle and mysterious agencies, atmospheric and terrestrial, of which we know so little except from their morbific effects. He is essentially a Materialist in all that pertains to pathology. Hence his inordinate confidence in what he considers the proper line of treatment, and his contemptuous disparagement of all who trust much to the *vix medicatrix naturæ*. The reader might often believe, from the unwavering nature of his author's assertions, that medicine was almost a perfect science; and yet, a few pages further on, he discovers a lamentation over the utter uncertainty that still prevails on the nature of some of the most wide-spread maladies. At one time, the fancy strikes him that, if there could only be a M. Bouillaud in every region of the world, the whole troop of acute diseases might be *chassé* from the surface of the earth, or at least brought under the dominion of science, and the clear light of unerring truth would be spread over every spot in the wide domain of medical enquiry. At another, he finds himself with his author plunged in the most perplexing darkness and confusion.

But it is time to proceed to an examination of the specific contents of the work before us; and we shall begin with the beginning. The first volume opens in these words:—

"Medicine is unquestionably in the first order of those sciences which, in modern times, have undergone that fundamental transformation invoked by the illustrious Chancellor Lord Bacon. It is indeed a magnificent spectacle for the philosophic observer to contemplate how, under the supreme empire of the law of progression, the sciences in general, and medicine in particular, accomplish, in spite of obstacles that are constantly springing up, the great work of their evolution. It would be too long to review all the successive stages through which the science of diseases has passed before arriving at the *new era*, that is to say, at the era of precision and exactitude. We must content ourselves with some rapid observations upon this very important subject.

"It is proper to render justice to the labours and discoveries of medical antiquity, and we should, without doubt, ever respect the almost divine name of Hippocrates, and bow before the lofty and vast genius of Galen. But our respect and admiration for the ancients must not, in any way, approach to idolatry, and hinder us from rendering justice to the moderns. Thus, then, without forgetting to honour past ages, let us also dare to praise the grand epoch in which we live, the true golden age of medicine and of the other natural sciences. To propose for our imitation, in the present day, the Hippocratic school of medicine, is about as reasonable as if we proposed for our model in physics and chemistry, the physics and the chemistry of Epicurus, Aristotle, or of Thales. The human mind does not thus move backwards, and our modern Hippocratists are at least twenty centuries behind their age! Who does not see, at the first glance, that Medicine—as long as she was deprived of the light supplied by *post-mortem* examinations, and unacquainted with the exact methods of investigation derived from the study of physics and chemistry—could be little else, if I may use the expression, than a scientific abortion bereft of every sense? Now this was very nearly the sad condition of ancient medicine!"

Without even so much as alluding to the writings of any medical men between the age of the Greeks and Romans and the commencement of last century, M. Bouillaud then with rapid wing comes down to the period when Morgagni "erected the first great monument to the new science of medicine," by the publication of his admirable work *De Sedibus, &c.* But it is to his own immortal country that belongs, we are told, the glory of having established the new school; and it is, our author thinks, a circumstance worthy of notice that this medical regeneration was effected "at nearly the same period at which, working for the benefit of the whole world, she (France) gave birth to a political revolution, which all future ages will remember with gratitude, and without which every sort of scientific progress must have been deprived of the liberty necessary for its fulfilment." Forthwith, we are favoured with a long analytic review of the labours of Pinel, and more particularly of those of Bichat—"he who with an eagle's flight traversed, in the course of a few years, the entire field of medical research, and, like an Alexander of a new stamp, achieved the conquest of the whole scientific world,"—and are then brought down to the time of Broussais, of whom our author has ever been a most ardent disciple. The subsequent career of medical science is thus portrayed:

"From the time when the doctrines of Broussais were predominant, medicine has steadily continued the course of its discoveries, and has been acquiring a continually increasing character of precision and exactitude. Favoured by happy circumstances, this science, thanks to the labours of men whose names will be cited in the course of this Nosography, has, during the last twenty years, made more *real* progress than she had ever accomplished in the same space of time, and the conquests, with which she has enriched herself, have been achieved in all the divisions which compose her vast empire. Medical philosophy has been systematised (formulée). The old methods of observation and research have been perfected, at the same time that new ones have been invented. By the united help of all these means, and above all by the clear light of the (properly called) accurate methods, physical and chemical, Medicine in our days has succeeded in discoveriug a good many diseases, hitherto overlooked, and it has, at the same time, more accurately determined the nature and true character of the greater part of those which were already known."

M. Bouillaud proceeds to detail some decisive conquests which he has achieved himself, within the last fifteen years, by following in the steps of his great teacher; he alludes more particularly to the discovery of the frequent existence of Endocarditis in Rheumatism; of the blowing and other peculiar sounds in the arteries when the blood is poor and watery, as in Anæmia, Chlorosis, &c.; and of the new formula for extinguishing inflammation by bleeding *coup-sur-coup*, &c. His grand object, he goes on to inform us, is to establish *unity* in medical doctrines, as it exists in those physical sciences which have attained the highest degree of exactitude. "This great work once accomplished, there will henceforth be no more merely routine medicine, and our science shall then have its code, like that of jurisprudence." He seems full of ardent hope upon this subject, being strongly impressed with the idea that the department of the diagnosis of diseases has already been brought to a truly marvellous degree of exactness, and that, when we have once discovered their natural seat, we can scarcely have any difficulty in employing the appropriate means for their cure. Something has gone wrong with the machine. Find out the seat of the mischief at once; be prompt and vigorous in applying the proper remedy; and, in the course of a day or two, all will be right as before. He over and over again appeals to the astonishing success which has attended his practice for the last twelve or fifteen years; and, upon this ground alone, he utterly rejects the idea adopted by some recent writers (Louis among the number) that the duration and mortality of diseases are often but little affected by the line of treatment that is pursued. Need we say that he is any thing but favourable to the *expectent* mode of treatment under almost any circumstances? If an enemy be in the camp, why not expel him at once, if you have the means? all delay but serves to make his expulsion the more difficult.

In the treatment of Inflammatory Diseases, he does not hesitate to affirm that his favourite plan of bleeding *coup-sur-coup* is omnipotent (*toute-puissante*) for their cure, reducing their mortality almost to zero! He illustrates his peculiar doctrines by the following comparison, that is, it must be confessed, more lively than convincing:

"Therapeutics is, at the bottom, an art which approaches to that of war; it is a sort of warfare against diseases. Now, just as is the case with the art of war, so that, which consists in combating diseases, ought surely to be subject to precise and accurate rules; and therefore the tactics of therapeutics, if I may venture to use the expression, must be reduced to certain exact formulas, in the same manner as military tactics are. Moreover the general principles of therapeutical tactics must be made to accommodate themselves, and bend to the different circumstances, from the operation of which various maladies are developed. In acute, violent diseases, which compromise in a greater or less degree the life of the patient, it should be the first principle to act with the greatest possible promptitude, and to oppose to the disease, that is to say to the enemy, means which surpass it in strength and rapidity. The means generally used until of late years were much less active than the intensity and rapid progress of the disease required. These means also failed in a great number of cases. It is to avoid similar checks and defeats that, in our practice, we have acted with more force and rapidity than our predecessors have usually done; and, thanks to heaven, success has crowned our efforts beyond all our hopes."

M. Bouillaud is naturally very much annoyed that scarcely any of his *compatriotes,* who have written on the subject of fevers and inflammation since the first announcement of the *nouvelle formule,* have even so much as taken notice of it. M. Martin Solon, in the Dictionary of Practical Medicine and Surgery, does nothing more than *dire le plus petit mot* upon the subject. M. Magendie is still more discourteous; for he does not hesitate to affirm that experience has condemned the practice of our author, and he goes so far as to insinuate that M. Bouillaud is often incorrect in his diagnosis: *il est fort douteux que ce soit toujours la maladie qu' on jugule*—the last word alludes to the strangling or extinguishing effects of bleeding *coup-sur-coup.* M. Andral has expressly said, in the second edition of his Clinique Medicale, that there are very few cases indeed in which an inflammatory disease can be suddenly removed by blood-letting, however copious and frequently-repeated. The same sentiment is expressed in his edition of Laennec; and in his late work on Hæmatology, he does not even deign to make any allusion to a practice which, we are over and over again assured, has already saved the lives of thousands. According to M. Andral, "it seems that, whenever the blood has begun to be charged with the excess of fibrine, it requires a *certain time,* whatever is done in the way of treatment, before this disposition can be removed." No one, replies our author, will deny the truth of this assertion, that a "certain time" is required to effect a cure; but this is not the point at issue. M. Andral cannot be ignorant that the new formula has not only diminished the amount of mortality from inflammatory diseases, but has also very materially abridged the duration of each case. Lastly, M. Louis has been equally unjust and illiberal; for, without giving the results of M. Bouillaud's practice, he does not hesitate to condemn the treatment employed by him: "we cannot strangle (*juguler*) inflammations, as some persons are too often pleased to say. Bloodlettings are not to be multiplied in the vain hope of obtaining this imaginary result." All this must be very mortifying to our author's vanity and pride: it is only to be regretted that he should so pertinaciously adhere to dogmas in practice, the fallacy of which it would not be difficult to prove from the pages of his present work. To us it is satisfactory to find that the opinions which we have uniformly held respecting M. Bouillaud, and which, during the last ten or twelve years, we have so often expressed without reserve in the pages of this Review, are fully confirmed in every particular by the express declarations of some of his most distinguished *confrères* in the existing school of French medicine.

There are many other subjects introduced and discussed in the Prolegomena; but our limits utterly preclude our noticing them now; and this is the less to be regretted, as they are all subsequently brought, at greater length, under the consideration of the reader in the special divisions of the work. We shall therefore now, without any further delay, proceed to a rapid analytic review of its chief contents; and, first of all, the subject of the nosological arrangement, or Classification of Diseases, adopted by our author, deserves a brief notice. He makes scarcely any reference to the labours of those who have preceded him, in this department. Pinel is the only one whose system is mentioned; and his "nosological pentateuch," as our author calls it,—1, essential or primitive fevers; 2, phlegmasiæ; 3, hæmorrhages; 4, neuroses; and 5, organic lesions—is thus summarily disposed of:

"Content with having criticised the doctrines of his predecessors, and substituted his famous code for more ancient systems, he troubled himself very little with formularising (*formuler*) any new doctrines on the nature or mechanism of diseases. Proceeding after the manner of naturalists, as he often complacently remarked himself, it was enough for him to describe what he called the external characters of diseases; and, conformably to this method, he described his *essential* fevers without formally attaching them to any special organs, in other words, without *localizing* them; and thus he incurred the reproach of being an *ontologist*, and too often shewed himself unfaithful, even in principle, to the fundamental doctrines of the medical school, whose founder was Morgagni." So much for the system of the celebrated author of the *Nosographie Philosophique*. Let us now see what is proposed in its stead, in the pentateuchal or five-volumed Nosographie Medicale of M. Bouillaud.

He starts with the assumption that "a truly philosophical and rational classification of diseases must rest upon the *nature* of these diseases; every nosological edifice, founded on any other basis, is necessarily unstable and fragile." He admits that a good deal remains to be done, before it can be said that we really know or understand the real nature of many maladies; still he thinks that our knowledge of medical etiology is sufficiently accurate to warrant the classification of all diseases under the following three heads or families:—

"The *first* family is composed of organico-vital or chemico-vital diseases, which essentially affect the chemical constitution or (as it is called) the internal structure of parts. The *second* comprehends such diseases as are purely dynamic, the Neuroses or lesions of the vital or nervous powers. To the *third* belong all the changes which occur in the purely physical, anatomical and static conditions, or, in other words, in the external structure of parts." But we must enter a little more minutely into particulars; because the nosological classification of our author, professing to be built upon the physiological nature of diseases, forms a key, as it were, to the discovery of his therapeutic principles. His primary three-fold arrangement is divided into, and made to comprehend, 12 classes; of which the first five belong to the first and second great families, and the seven last belong to the third family. Here is the Synoptical Table of these twelve classes, which, be it remembered, embrace surgical as well as strictly medical diseases.

I. Fevers and Inflammations (Pyrexiæ).

II. Affections consisting in a defect of excitation or vital action.

*Appendix to the first* 2 *Classes.*

Excess and Defect of Hemat[illegible]sis.

III. Ataxiæ of the Nervous Centres.

IV. Miasmatic and Virulent Diseases.

V. Heterotrophies, Heterocrinies, and Heterogenies, of a non-inflammatory nature.

VI. Extravasations in general, and Hæmorrhages in particular.

VII. Solutions of Continuity, and Abnormal communications.

VIII. Displacements and Deviations.

IX. Abnormal Adhesions, Connexions, &c.

X. Changes of Extent, Volume, and Capacity.

XI. Foreign and retained substances.

XII. Changes in the configuration.

The 1st Class, including the Pyrexiæ or Fevers and Inflammations, is like Aaron's rod; for it nearly swallows up all the rest, according to M. Bouillaud's views of the matter: no less a portion of his work than three volumes and a half out of the five being occupied with a consideration of the diseases in question. Our readers may therefore expect to find an immense assemblage of maladies classified under the head of Inflammations and Fevers. The Class is subdivided into three orders. These are —I. The genuine phlegmasiæ and continued fevers; including not only such diseases as are usually so denominated, but likewise the whole tribe of Cutaneous diseases, from small-pox and scarlatina to prurigo and scabies, as well as glanders, tuberculization of the lungs, and cholera-morbus! II. Irritations properly so called, and Intermittent fevers; or active Neuroses (hyperneuries). Besides agues, this order contains various convulsive and spasmodic diseases, such as asthma, neuralgia, epilepsy, chorea, mania, &c. III. The third order (which is complementary to the two preceding) comprehends the various products of inflammatory and irritative disease; such as active serous effusions, hypertrophies of different organs, and various morbid degenerations of structure.

The 2nd Class is divided into two orders; the distinctive character of the one being the "complete abolition or simple diminution of the nutritive life common to all parts;" and that of the other, the "complete abolition or simple diminution of the forces presiding over the special action of different organs." The former includes gangrene, local asphyxia, and atrophy; while in the latter we find all passive neuroses, paralysis, amenorrhœa, dysmenorrhœa, &c.

The chief members of the 3rd Class, or the *Ataxic Affections* of the nervous centres, are 1, the various irregularities in the sounds and pulsations of the heart; 2, irregularities, incoherence in the co-ordinated movements of locomotion, articulation, &c; and 3, irregularities, incoherence in the moral and intellectual functions.

The 4th Class is devoted exclusively to the reception of the three species of Typhus—European, American (the yellow-fever), and the Oriental (the plague).

The 5th Class is somewhat akin to the Cachexiæ of other nosologists, and comprehends scrofula, cancer, scurvy, diabetes and several other urinary affections, entozoa, &c.

The 6th Class contains the numerous varieties of hæmorrhage, from apoplexy of the brain to the formation of petechiæ, vibices, and scorbutic ecchymoses.

We need not particularise the contents of the remaining six classes of this extraordinary nosological table, as they relate chiefly to surgical maladies, congenital malformations, and so forth; nor have we any intention to examine critically its merits and demerits. We should think that there can be but one opinion on the subject; viz. that it is perhaps the very worst classification that ever entered into the head of learned doctor to propose. The identification of continued Fevers with genuine Phlegmasiæ; the divorcement of the different forms of Typhus from those of Typhoid fever, and the establishment of the former in a class by themselves, as if they were diseases altogether distinct and dissimilar from all others; the dissociation of Intermittent and Remittent fevers from those of a continued type, and their arrangement among the Neuroses; the strange amalgamation of bodily and mental disorders in the 3rd Class;—these are a few out of the many glaring blemishes that deface the nosological scheme under review.

As the present article shall be confined exclusively to the consideration of our author's views on the very important subject of Fevers, it will be obvious that the 1st and 4th Classes of his Nosology are those which will chiefly occupy our attention. The great aim and object of all his remarks is to show that the so-called *essential* continued fevers are really and truly inflammations, either simple or complicated with a septic (typhoid) element; and this position he is now more than ever anxious to establish, seeing that "quite recently several authors of authority, (Andral, for example, *Rev.)*, retracing in some measure their steps, have endeavoured again to distinguish into two separate classes diseases which they formerly regarded as identical in nature." If a confident and unwavering hardihood of expression—founded we verily believe, on a conscientious conviction of the truth of his observations—will ever make converts, few, who read the present work with an orthodox (query, popish?) faith, would escape becoming Bouillaudists; unless, indeed, the inordinate zeal of the writer to establish his own views suggested the idea of some weakness in the cause that he was advocating.

The subject of Inflammation in general is discussed at great length. All that we can attempt to do is to select a few passages, which seem to explain the peculiar views of our author on the important question, how far inflammatory and febrile actions are to be considered as identical in their nature and phenomena.

"Considered in itself, in its essential nature, Inflammation is *one*, and constitutes a *single* species; but, considered in relation to its complications, its causes, its degrees, and extent, &c. it exhibits numerous differences, and may be divided into a great number of species. As to its complications, that which is most worthy of attention, is the *septic*, *putrid*, or *typhoid* condition. What we have said of Inflammation in general is strictly applicable to *Continued Fever* in particular; a disease, which is nothing more than the result of a primary or secondary plegmasia of the sanguineous apparatus." The supervention of the *typhoid* condition, in the course of an inflammatory disease, is attributed to the absorption into, or to the generation in, the system of certain morbid matters, which poison and infect the whole mass of blood. When this is the case, "there exists (to use the lucid description of our author) a sort of two-fold general state, composed of inflammatory re-action on the one hand, and a septic infection of the circulating fluids on the other—elements, so different from each other, that the latter is characterised by a softened condition of the coagulum of the blood and the absence, or at least extreme softness, of the buffy coat, which is invariably present in all pure and simple inflammations." He describes this typhoid complication as being to the entire system what gangrenous decomposition, in consequence of local inflammation, is to a part: "in the latter case, there is, so to speak, a *local* typhoid fever; while, in the former one, there is a *general* typhoid fever." In the same spirit, he tells us, that "the general state known by the name of *fever* is for the entire sanguineous system, what the inflammatory state is for that portion of the vascular system in a special diseased organ. In one word, acute fever is a general phlogosis of the vascular system, and inflammation of an organ is a *local fever.*"

We come now to consider another question; and, as it is one that has important practical bearings, the reader, we trust, will be pleased to excuse the inconvenience of the occasional repetition of opinions, and, it may be, of nearly the same language.

"Is the division of Fevers into idiopathic or essential and symptomatic natural or legitimate? This grave question, which for the last 25 years has been discussed under every possible aspect, is not yet finally settled in the opinion of some physicians. Assuredly it would have been so, had the facts been presented to them by public lecturers and teachers with that perspicuity, force, and good faith, which constitute the very soul of science in general, and of medicine in particular. Unhappily this has not been the case. For the last 20 years that the author of this work has studied the great, the immense, question of fevers, he has neglected nothing that might contribute to the triumph of truth, and he still continues his devoted exertions; too happy if he might at length succeed in dissipating, past all chance of return, the uncertainties which still prevail on a subject of so much importance, and in introducing order and light, where as yet we meet with only darkness, confusion, and chaos."

After this flourish of words, the reader is informed that all (the diseases usually considered as) essential fevers have an element or character in common; to wit, that state of inordinate excitement of the system, which is indicated by increase of *heat*, and of the force and frequency of the cardiac and arterial pulse:—otherwise, says our critical author, the term *fever* would be utterly inappropriate! This is a mere etymological quibble, that is utterly unworthy of any practical writer. But M. Bouillaud does not appear to regard it so. Starting from it as a point, on he goes in the most amusing style of self-complacency and self-gratulation, pitying his poor *confreres*, who are still groping their way in darkness, while all is sunshine along his path. After alluding to the discordance of opinion that still prevails on the subject of the nature and cause of fever among the physicians of that metropolis, which is the centre of civilization, the eye of the whole world,(!) he thus modestly tells them how everything may be rectified:—

"What must be done to put an end to the truly afflicting spectacle of such contradictory doctrines, this veritable anarchy that prevails in the midst of the foremost medical school of the universe?—an exact knowlege of facts, and an equally exact nomenclature, *i. e.* a nomenclature that might be a faithful representation of the seat and sort of lesion, simple or complicated, single or multiple, which characterises each of those morbid conditions that have been studied under the name of essential continued fevers. But then we must remember that, to understand pathological facts aright, we must observe them under all their aspects, and with the aid of all those exact methods with which the majority of medical men are as yet but very imperfectly acquainted. It is by following this method that the author of the present work has at length succeeded in solving, as far as the existing state of science will admit, the various questions appertaining to pyretological science. He has had the satisfaction of seeing his doctrines adopted by all the students that have duly attended his clinical instructions. He believes that they will be generally adopted by the whole of the rising medical generation, provided they be taught in a worthy and suitable manner in lectures and books, as well as at the bed-side of the patient. But he is quite aware of the difficulties which oppose *unity* in the mode of instruction, and, if he regrets at having still so much to contend with for the triumph of what he *knows* to be the truth, this is not from any feeling of vanity, but solely and altogether from the sound and deep-felt interest he has in the welfare of humanity. Not a year passes but thousands of persons, affected with the so-called essential fevers, owe their lives to that treatment which he has been so fortunate as to propose; for these fevers, of a non-miasmatic origin, are invariably veritable inflammations at first."

It will naturally be asked, inflammations of what part, texture, or system?—Of the sanguiferous system, the blood and the blood-vessels, replies M. Bouillaud. In accordance with this view, simple fever is described and treated of under the head of the Phlegmasiæ, and in the chapter, entitled, "Of inflammations of the sanguineous apparatus in general, and of each of its divisions in particular." One of the opening passages in this chapter runs thus:—

"This phlegmasia—inflammation of the internal membrane of the sanguineous apparatus, or angeio-carditis*—so long unknown, is nevertheless one of the most frequent of all, whether it arises primarily and idiopathically, or consecutively to local phlegmasiæ. When simple, exempt from all foreign complication, it constitutes the pure inflammatory or *angeiotenic fever* of Pinel, the *synochus* of other authors. When complicated with a septic, putrid, or typhoid element, it is the fever that has been called *putrid, typhoid,* or *adynamic.*"

Without noticing the inflammatory lesions of the heart and blood-vessels *alleged* to be found in all fatal cases of Synochus, let us ascertain what our author says respecting the condition of the blood itself, and see how far his dicta correspond with the hæmatological observations of Andral and some other of the leading physicians of the French metropolis. M. Bouillaud affirms that the blood is uniformly strongly buffy and cupped, exhibiting all the characters that we see in cases of pneumonia and acute rheumatism. It is truly surprising, he says, that distinguished authors, Pinel for example, should assert that in inflammatory fevers the appearances of the blood drawn are very variable, and do not present any well-marked diagnostic features; and then he adds, with most amusing simplicity: "whenever we meet with the buffy coat of the blood, such as I have described, we may confidently assert that there exists a general inflammatory diathesis, a phlogosis of the sanguineous system, a *sanguine fever.*"

Before concluding his remarks on the state of the blood in Synochus, we are favoured with the following very satisfactory (!) mention of the recent researches of MM. Andral and Gavarret on this subject.

---

* The appellation of *angeio-hemitis,* proposed by Professor Piorry, is, in our author's opinion, preferable; as it points to the state of the blood, as well as of the blood-vessels.

"The blood, in cases where the characters described above are present, contains a larger proportion of fibrine than exists in a state of health. The buffy coat is in a great measure composed of this fibrine. Whence this excess of fibrine? MM. Andral and Gavarret unhappily leave us in a state of complete ignorance on this point. May we not attribute it to a sort of pseudo-membranous secretion from the inflamed membrane (the inner coat) of the blood-vessels and heart?"

Whether any of our readers will be better able to diagnosticate a case of fever after reading the following observations, we cannot determine; we must leave it to themselves to say.

"How shall we establish the differential diagnosis between a primitive, idiopathic, *essential* fever, and one that is secondary, sympathetic, and symptomatic. The thing is by no means so easy, even in the present day, as many physicians imagine; and it is not surprising that our predecessors, with their imperfect methods of observation, should have quite mistaken certain kinds of phlegmasiæ, and have designated, by the epithet of *essential*, fevers that are symptomatic of these very phlegmasiæ. I have often witnessed the very opposite mistake committed, and seen, for example, attributed to a phlegmasia of the glands of Peyer, a fever that was quite independent of it; I have seen a case of small-pox, before the eruption had made its appearance, mistaken for one of typhoid or entero-mesenteric fever, &c. &c. It is only by a profound acquaintance with the positive signs, by which the various local phlegmasiæ are indicated, that we can hope to distinguish the two species of fever (essential and symptomatic) from each other. When these signs are *really* awanting, then we must acknowledge that the fever exists by itself, *i. e.* independently of any local inflammation, and is strictly and truly idiopathic; but it should always be borne in mind that some deep-seated local phlegmasiæ may exist with scarcely any appreciable manifestation of symptoms. It is told of a celebrated observer, that on one occasion, being exceedingly annoyed at not finding any visceral lesion in a fever patient, whom he believed to have had a *gastro-enterite,* he stuck the point of the scalpel into various parts of the corpse, when, lo! a quantity of purulent matter gushed out from a wound made in one of the thighs. This case was certainly not favourable to the doctrine of the essentiality of fevers; but it shewed by the double error committed, on the one hand, that a continued fever may be attributed to a phlegmasia which does not exist, and, on the other, that the phlegmasia, which really caused the fever, may be entirely overlooked. Many years ago, I witnessed a very similar case. A woman died of continued fever. No trace of any visceral lesion was discoverable upon dissection; and the physician was much puzzled to find the *point de depart* of the fever, when at length he detected a vast abscess situated immediately in front of the vertebral column."

It is scarcely necessary to point out the absurd mistake here of confounding together Synochus and Hectic fever, as if they were merely different degrees of the same morbid action. Our author, indeed, afterwards tells us that the latter fever, whether it be primitive or only symptomatic, is never the simple consequence of an angeio-carditis, but that there is always a greater or less degree of vitiation of the blood from the admixture of purulent matter. Surely such a humoral lesion is not present in cases of synochus; and, if not present, why not dissociate, it may be asked, the one from the other?

It is amusing to observe how very nearly, every now and then, M. Bouillaud is becoming an essentialist, in spite of himself. When speaking of the causes of synochus, he enumerates, first of all, over-feeding, excessive exercise, exposure to great heat or to alternations of temperature, and then he proceeds as follows:—

"The continued fever, which precedes the first period of acute cutaneous eruptions, specific and contagious, and of *some other analogous affections,* (the Italics are ours, *Rev.*) is probably attributable also to the introduction of miasms, virus, or subtle foreign principles, into the sanguineous system. Under this aspect, the fever in question may be associated with the different species of inflammatory or angeiotenic fever. But then we must remember that these principles, of whatever kind they may be, impress a peculiar stamp and special physiognomy upon the fever, in consequence probably of some specific action upon the blood and, it may be, the nervous system also. We must wait for further researches before we know what to make of this point of etiology."

As a matter of course, the treatment of simple inflammatory or angeiotenic fever is one of the easiest things in the world. We have only to withdraw from the mass of blood a certain amount (une proportion donnée) of its plastic elements, then prevent its rapid reformation by keeping the patient upon a spare diet, and dilute the remainder by administering cooling diluents: *voila tout!*

So much for Synochus, or Angeio-carditis when it is "simple, franche et legitime;" let us now hear what M. Bouillaud has to say about "angiocardite typhoide, c'est a dire avec une complication d'un etat putride ou septique (adynamique)." This species of continued fever is said to be *often* (we had fancied that it was *always*; otherwise why call it angeiocarditis? *Rev.)* associated with a local phlegmasiæ; but then this phlegmasia has become the focus of a septic infection: it is from the existence of this double morbid element that the peculiarities of the disease arise. "When Synochus or inflammatory fever is idiopathic, the typhoid phenomena may be the effect of septic matters or miasms received from without or generated within the vascular system, the inflammation of which must have terminated in a genuine suppuration, and have given rise to what, of late years, has been denominated *purulent infection*"

We have no intention of following M. Bouillaud through the "tableau fidele" which he professes to have given of the symptoms of typhoid angeio-carditis, or *Synochus putris*; they are exactly those usually specified as attending cases of *typhus gravior*. His description of the blood in this disease is worthy of notice, as evidencing a desperate clinging to a favourite doctrine in spite of the most damning proofs of its utter fallacy.

"I have previously said that the presence of the typhoid element exercises a marked influence upon the accompanying inflammatory element: this influence is especially obvious in the state of the blood. In truth, we must no longer expect to meet with the characters of inflamed blood, such as we have described above. If the typhoid condition be decided, or if it has continued for some time, the coagulum is soft, *dissolved,* of a blackish colour, and generally adhering to the walls of the vessel in which it is contained; it exhibits either no buffy coat at all, or this is remarkable for its softness and serous infiltration." These characters, we are then gravely informed, are so very different from those of inflammatory blood, that nothing is more easy to any one who has clinical experience than to pronounce at once, after a simple inspection of the blood drawn, whether the case be one "d'une fievre franchement inflammatoire ou d'une fievre avec complication septique ou typhoide." We should think so, indeed. Here again, the notice of MM. Andral and Gavarett's researches upon this point is most expressively (!) short. All that we learn respecting them, is that "in fevers or phlegmasiæ (is not the introduction of *phlegmasiæ* here a very wilful and most unwarrantable interpolation of M. Bouillaud himself?) with typhoid phenomena, the proportion of the fibrine in the blood is diminished,—the very reverse of what exists in active inflammations." Why our author should so rarely and imperfectly allude to the hæmatological investigations of M. Andral, and so designedly slur over their cardinal and most essential conclusions as to the marked difference of the blood in genuine Phlegmasiæ and in Fevers, is very strange. Is it retaliation for neglect of the *nouvelle formule?*

So decided a humoralist has M. Bouillaud become of late, that he even finds fault with other writers for ultra-solidist opinions.

"If," says he, "anything can astonish us now, it is the fact that Pinel should have considered as purely imaginary, and as utterly incompatible with life, a certain degree of putridity of the blood. Nothing is more positive or more real than this important alteration of the circulating fluid;—an alteration that results from a sort of poisoning of the living fluid by putrid principles, in the same manner as intoxication results from the introduction of alcoholic principles into the torrent of the blood. The typhoid intoxication, if we may so speak, being in truth the effect of a lesion of the blood, the term *adynamic,* applied to the fever in which it exists by Pinel,—because there is, as he said, *une atteinte profonde portée directement aux forces musculaires*—suggests no idea of the true nature of the disease."

Lastly, it deserves to be noticed that our author now expressly cautions his readers against mistaking certain post-mortem appearances as evidences of inflammatory action:

"In consequence of the rapidity with which the corpse of a patient, who has died with typhoid symptoms, runs into decomposition, we must be on our guard, in necroscopic examinations, not to refer to any vital lesions certain alterations which may be the simple consequences of cadaveric decomposition. Such are, among others, certain *ramollissemens,* certain rednesses of the lining membrane of the heart and arteries, certain productions within this apparatus, as well as underneath the mucous membranes, &c."

Such is the sum and substance of the section of the present work that is devoted to the consideration of inflammatory and of typhoid Angeio-carditis; in other words, of the Synochus and Typhus of Cullen, if we can judge from the narrative of their symptoms, apart from all speculation as to their nature or seat. But it would be giving a very imperfect notion of our author's views, if here we closed our remarks upon this subject; as much yet remains in other sections of this Nosography that unquestionably appertains to it. Passing over a great variety of phlegmasiæ, including all the contagious exanthemata as well as other cutaneous eruptions, we at length (Vol. III. p. 92) come to the well-known theme of *Entero-Mesenterite,* under which appellation M. Bouillaud has hitherto been in the habit of treating of the disease, which has been at different times called putrid, malignant, typhoid, ataxic, adynamic, &c. fever. After a variety of details, which it is not necessary for us to notice, the following passage arrests our attention:

"We now add," says M. Bouillaud, "the epithet *typhoide* to the term *entero-mesenterite;* as, in truth, this phlegmasia—in consequence of its seat in an organ which always contains certain fætid and fermentable matters—is more than any other apt to give birth to this class of phenomena. Nevertheless, in its early stage, this phlegmasia is exempt from all typhoid or septic elements, and indeed the supervention of the typhoid symptoms may be almost always prevented by the employment of active antiphlogistic measures at first."

Its essential and pathognomonic character, it is to be remembered, is an inflammation of the Peyerian and Brunnerian glands of the mucous coat of the intestines.

Having given a very minute account of the anatomical lesions of the intestines and mesenteric glands, our author proceeds to describe some other morbid appearances that are often met with. "We should be much deceived if we were to believe that the alterations hitherto described are the only ones discoverable in the alimentary canal in patients who have died of the *so-called essential fevers* of Pinel, which by certain writers have been all classified together under the general term of *typhoid fever* or *typhoid affection*. In an immense majority of cases, the stomach and the large intestine will be found to exhibit marks of inflammation more or less severe and extensive." But then, with a most diverting inconclusiveness, he seems to imply that it is only one sort of Typhoid Fever to which the preceding remarks apply: "I have formally concluded, from my observations, that a veritable *gastro-adynamic* or *bilioso-putrid* (here the regular terms of Pinel are employed) has, for its essential and characteristic anatomical lesion, an inflammation of the mucous glands of the small intestine, and especially of those situated in the lower end of the ileum." The secondary or consecutive lesions of this entero-mesenteric fever are said to be various; we shall only notice those that appertain to the sanguineous system. They are thus enumerated—redness of the internal membrane of the large vessels, as of the aorta, pulmonary artery, &c.; notable puffiness of the valves, and a softening of the muscular substance of the heart; a black dissolved state of the blood; softness of the fibrinous concretions sometimes met with in the cardiac cavities. In a subsequent page, we read the following description of the characters which the blood exhibits in this disease: "In the early stage, when the fever exhibits itself under an inflammatory aspect, the coagulum presents a certain degree of retraction (retrait); its consistence is nearly normal; its surface is sometimes covered with a general or partial buffy coat; but the blood is never, unless some complication is present, *franchement inflammatoire*, as in a pure simple phlegmasia. * * * In the second and third stages of the disease, when it assumes the typhoid or putrid (adynamic of Pinel) type, —*i. e.* when the septic are superadded to the inflammatory phenomena— the state of the blood is what has been already described in our first volume," under the head of typhoid Angeio-carditis. Then, M. Bouillaud had a hit at M. Pinel for his neglect of humoral pathology; now, M. Chomel comes in for a gentle rap: "For the last 13 years, I have treated more than 500 cases of *typhoid fever*, and the blood has uniformly exhibited the characters we have described to be present in the different stages. How then comes it to pass that a clinical professor, who has published his lectures on this disease, should assert that the blood drawn from a typhoid patient does not exhibit any appreciable alteration, except in a small number of cases?" We may fairly ask, what are the morbid changes that are specified by our author himself to be present in the first stage of typhoid fever. From his own description, it is utterly impossible to make out; there is such a confusion and jumbling together of different things on the one hand, and such a forcible divorcement and separation of the same, or nearly the same, things on the other. M. Bouillaud here, as on the former occasion, when treating of angeio-carditis, slurs over and even mis-states some of the leading facts and conclusions connected with the recent hæmatological enquiries of M. Andral.

With respect to the general *symptomatology* of entero-mesenteric fever, it seems to be identically the same with what had been described in a previous volume as indicative of typhoid angeio-carditis; with the simple exception that, in the former, certain abnormal symptoms—diarrhœa, tenderness over the right iliac region, &c.—*may be* (for it is acknowledged that they are not always) present. That the *Diagnosis* of the disease by the physicians of Paris is any thing but easy or to be depended upon, is abundantly obvious from the following account by one of themselves of the blunders often committed in that seat of learning and centre of medical knowledge: "I have seen," says M. Bouillaud, "I regret to say it, physicians of established reputation, but who have not been educated in the principles of exact medicine, mistake for a case of typhoid fever one of peritonitis and even of pneumonia. On how many other occasions, have I not witnessed a clear case of typhoid fever entirely overlooked; and, on the other hand, a case of simple gastric or intestinal derangement set down as a specimen of fever!" After this acknowledgment, who will gainsay the fairness of those strictures which, for the last ten years, we have felt it necessary to make upon the untrustworthiness of much of French medical literature upon practical subjects?

It is certainly passing strange that, when M. Bouillaud treats of the diagnosis of *entero-mesenteric fever*, not even a hint is made of the very disease, which of all others we should have thought would be most apt to be mistaken for it—we mean, the *angeio-carditis* of which we have already heard so much. Does he really suppose that his readers are so very forgetful, or that they peruse his work with so little attention, as not to be struck with this remarkable oversight (to call it nothing else) on his part? He evidently feels sore at the incredulity of many of his brethren as to the correctness of his own diagnosis in what may be called his *pet* diseases, in spite of his confident and ever-repeated assurances of his almost unerring certainty. Witness the following passage:

"I know that for the purpose of explaining why, in my clinical practice, the mortality of *real typhoid fever* (is this entero-mesenteritis, or angeio-carditis, or either of them?—we cannot tell.—*Rev.*) is found reduced almost to zero when the disease is treated in time according to my method, many persons have found nothing better to say than that the cases cured were not examples of typhoid fever. Happily for me, and more especially for my patients, this assertion is a most gratuitous insinuation. I can appeal to the impartial judgment of competent men who have followed my clinical visits, and under whose eyes I have dictated the diagnosis and prognosis in each case after an examination whose precision and exactitude are well known. Is it to me, in conscience, that such a reproach, such an insult, can be addressed? What! some one will say, do you cry out so sharply against others who accuse you of errors in diagnosis, and yet make this very charge against them? It is quite true; but be it remembered that I have not done so until after I had acquired the certainty of the error, and it is not assuredly in this manner that my opponents have acted towards me."

How liberal! how modest! how convincing! We have already said that the mechanism (to use our author's language) of the typhoid phenomena in entero-mesenteritis is supposed to consist in the absorption of putrid matters in the intestines by the ulcerated abraded patches on their inner surface. The supervention of the general or systemic typhoid symptoms is always coincident with that of local septic phenomena, evidenced by tympanitic distension of the abdomen, discharge of fætid stools and gas from the bowels, &c. These latter phenomena attest in the most striking manner the transformation of the *pure* inflammation into one that is putrid or even gangrenous.

"This circumstance," continues our author, "superadded to the conditions of *natural* septicity which occur in the inflamed organ, serves to increase the activity of the focus of infection; and whoever seriously reflects upon this union of causes of putrid infection, which do not exist to the same degree in any other phlegmasia, must cease to wonder at the circumstance that of all inflammations it is that of the mucous glands of the last convolutions of the small intestines, which is peculiarly apt to give rise to septic or typhoid phenomena. It would indeed be surprising, if it were otherwise; and it is a thing truly most satisfactory, that the researches of anatomy and physiology have led the moderns to the discovery of a phlegmasia so situated in a very great number of patients affected with putrid or adynamic continued fever, the *point de depart* of which had been so long unknown, and to which the term *essential* had been so unfortunately applied."

As to the exciting causes of true typhoid or antero-mesenteric fever, M. Bouillaud has absolutely nothing to tell us. He has rigorously examined 600 cases within the last 13 years, in the hope of arriving at some certain conclusions on this head; but hitherto without success. Vitiated air from the crowding together of masses of people may have occasionally some effect; but we must be on our guard, we are told, not to attach undue importance to this particular; for "how many are the patients that have assured us that they were well lodged and breathed a pure air!" Truly, M. Bouillaud is a most believing man, on some occasions. With respect to *contagion* having anything to do with the diffusion of the disease, he unhesitatingly determines the point in the negative.

After what we have said of our author's pathological views, we need scarcely dwell upon the treatment which he recommends. As a matter of course, bleeding is the sheet-anchor in the first or (alleged) inflammatory stage of the fever. In the second and third stages, the chief reliance should be placed on the use of demulcent, detergent, and antiseptic drinks and enemata, for the purpose of purifying and cleansing the abraded intestinal surface from the putrid matters adhering to it. The closing paragraph in the chapter on the treatment of the disease conveys the following most instructive advice:

"In the second and third (the putrid) stages of the disease, ought we to have recourse to the purgative method, as it has been proposed (formulée) of late years? I declare that I do not possess the necessary data for the perfect solution of this question. But I must from this moment confess that, after the results obtained from the use of this method, such as I myself know them, it seems to me preferable to keep to the use of mucilaginous diluent drinks, emollient poultices and injections, antiseptic medicines such as the chlorurets, external revulsives, and of some other means suited to the various complications of the disease."

We pity the medical man that is turned adrift to practise his profession, perhaps in military hospitals, on board-ship, or in some unhealthy locality, with such a miserable cargo of therapeutic directions; but ten-fold more are their poor patients to be compassionated.

Having thus ascertained the opinions of M. Bouillaud on two forms of *typhoid fever*, we shall now briefly examine what he has to say on the subject of *typhus;* which, as has been already stated, is considered by him to be a disease so totally distinct from the former as to be arranged in a class by itself. This class is the IVth, and is devoted to the description of "Miasmatic and Virulent Diseases in general, and Septic Diseases or Typhus in particular." Were we disposed to be very critical, we might, in all simplicity, enquire whether Ague and Remittent fever are not miasmatic diseases, not to mention the purely infectious exanthemata. But, without spending time on controversy, let us proceed to find out what our author has to say about Typhus.

First of all, he finds fault with all such vague terms as *typhus, plague, yellow fever, jail-fever,* and such like appellations. They have no precise meaning attached to them; it is therefore all but impossible to discuss their history. "Hence it comes that we know little or nothing about them; and the only plan to do, if we hope to understand their nature, is to throw overboard all the works that have been hitherto published, and have recourse anew to the book of nature. Ten observations, made with *exactitude,* on each of the above maladies, would do more to enlighten us than all that has yet been written upon them."

The first position that our author lays down respecting typhus and all other pestilential fevers, whether in Europe, Asia, or America, is, that in all cases without exception, there is a double morbid element at work—inflammation, and a septic or putrid infection. In some cases, indeed, the latter element predominates over the former from the very outset; but this is not always the case; for the typhoid condition may not be present at first, and may be only grafted upon the inflammatory, in the manner we have described in a former page, when treating of the history of entero-mesenteritis. His second position is, that there are three sorts or species of Typhus—the European, the American or yellow fever, and the Oriental or the plague. These three are not so much different diseases, as varieties of one and the same disease. None of their alleged characteristic features are uniform or constant. Buboes and carbuncles have been witnessed in some cases of typhus; and the black vomit is not invariably peculiar to the yellow fever of the new world.

With respect to the seat or nature of the three species of typhus, our author acknowledges that it must be sought for in the blood. But, in seeking to explain the changes that take place, he is continually falling into the most bewildering errors, in consequence of his mind being ever intent upon mixing up some of his own conjectures with the facts which he describes. For example, he asserts that all miasmatic poisons induce an inflammatory action in the parts with which they come in contact; and then, having settled this point in his own mind, he proceeds to talk very complacently of the invariable coexistence "des etats phlegmasiques" with "des etats de decomposition septique," in the different forms of typhus or putrid fever. He says that, as yet, we possess no satisfactory information as to the change that takes place in the mass of the blood in this disease. He calls it " a sort of gangrene of this fluid; for it is essentially the same change as that which gives rise to gangrenous decomposition in a case of local septic infection." In confirmation of this statement, he quotes the observations of M. Bonnet of Lyons, to the effect that the blood of typhus patients contains *hydro-sulphuret of ammonia*—a salt that is well known to exist in putrescent blood. He refers to what he had previously said concerning the state of the blood in typhoid angeiocarditis and entero-mesenteritis, as being applicable to the hæmatology of genuine typhus; and he expresses his astonishment that Pinel, Broussais, and several other writers (whom he afterwards calls *exaggerated solidists*), should have been so blind as, " in the midst of symptoms of the most flagrant septicity," to call in question the existence of an altered state of the blood—*ce grand element pathologique*—in patients labouring under typhus or adynamic fever. As he proceeds, he becomes quite a zealous out-and-out humoralist.

" It is not the blood only that exhibits alterations in typhus. As this fluid supplies every part and tissue of the body, and furnishes the materials of all the secretions, it is obvious that every part must be more or less morbidly affected. Among the most frequent alterations, I may mention the vascular injection of different organs, *ramollissemens* partly inflammatory, partly gangrenous, suppurations, sanguineous effusions, and extravasations, &c."

With respect to the Etiology of true Typhus, M. Bouillaud agrees with the general opinion; for we find him admitting, that " the essential cause of the disease is a putrid miasm diffused in the atmosphere, and acting on masses of persons crowded together in ill-ventilated situations." He recognises the contagiousness of the three species of Typhus; but adds that, in his opinion, the influence of this agency has been much exaggerated. His views on the subject of Contagion are evidently the result of a very imperfect examination of details. Indeed, the whole of this section of his work, that professes to treat of miasmatic diseases, is rather an unconnected commentary on the statements of certain authors than any regular exposition of the matter in hand. For example, he denies that any form of true typhus ever exhibits a remittent or intermittent character, and gives the following most convincing reason for his opinion:

" The element, that is common to intermittent and to continued fevers, is the excitation of the sanguiferous system: this element may exist either alone, or combined with a state of miasmatic infection. But in *continued* fevers, there is present, either in the sanguineous apparatus itself, or in different special organs, a veritable inflammation that keeps the febrile excitement under its dominion, and makes it assume the continued type which necessarily belongs to itself; whereas, in *intermittent* fevers, on the other hand, the febrile excitement is exempt from all such inflammatory complication: if this were not the case, the intermittent type would be absolutely impossible."

Our author seems to have forgotten all the while that he was treating of typhus, which, he had himself admitted, might exist without any inflammation at all!

The description given of the symptoms and treatment of typhus as seen in Europe, is about as absurd a thing as we have ever encountered. M.

Bouillaud evidently knows nothing of the matter from his own experience; and at length, after a good deal of most unnecessary verbiage, he is obliged to admit that there is not an atom of difference between typhus and many cases of his entero-mesenteric, or of his angeio-carditic, fever. "The symptoms of the former," says he, "are essentially those which I have described when treating of the septic forms of continued fever (is typhus not a continued fever?—*Rev.*), and of entero-mesenteritis. I will only remark, that there is in the last-named disease phenomena of local septicity and symptoms of general septicity, associated with those of inflammation. Now, *if* (!) typhus exists without being accompanied with the special inflammation of the mucous follicles of the ileum, it will not exhibit the local phenomena peculiar to this affection. In the contrary case, these phenomena will present themselves in a more or less decided manner."

The account given of the American Typhus or Yellow Fever is, as might be expected, very scanty and imperfect. Pinel, we are told, considered it not as a disease *sui generis*, but merely as one form of *gastro-adynamic* or *putrid bilious* fever; its peculiarities depending upon climate and other accidental causes. Tommassini was of the same opinion. M. Bouillaud flatly denies that the yellow fever (although he never saw a case) ever assumes a remittent, or intermittent type! The leading and most frèquent anatomical characters of the disease are inflammation and ulceration of the stomach and small intestines, the presence of a dark sanguineous fluid in the stomach, engorgement of the lungs, traces of inflammation of the arachnoid, &c. M. Louis, in his report of the Gibraltar Epidemic, stated that a yellow discolouration of the liver was the only uniform and characteristic lesion, not observed in other diseases! On the other hand, M. Rufz of Martinique assures us that this is only an occasional appearance. Not even a passing allusion is made by our learned (?) author to the writings of any English physicians, although, we need scarcely say, no men have seen so much or written so ably, on Yellow Fever as our countrymen. But M. Bouillaud knows nothing about them; perhaps he has not even heard their names. He regrets that he has never been able to institute any personal researches on the true nature of the disease; then doubtless all difficulties would have been cleared up. He tells us that the symptoms of Yellow Fever are essentially the same as those of Typhus, and thereupon he sends his reader back to what he had said on this head!

With respect to the question whether the disease be contagious, it appears that the majority of French writers are inclined to return a negative answer; and even those, who adopt the contrary opinion, are all in favour of greatly relaxing the existing quarantine regulations.

As to treatment, M. Bouillaud very summarily dispatches that subject. "Hitherto," says he, "nothing precise or rigorously exact has been established. We must look to the labours of writers yet to come, for accuracy; their researches must be conducted in such a spirit of exactness, that no serious objection shall be made against the results which they have obtained!"

We shall not follow our author through his equally meagre, and often incorrect, description of Oriental Typhus or the Plague; we shall wait till he has seen it.

Even on the more domestic, and therefore better known, subject of Intermittent Fevers, many of his views appear to us crude and most crotchety. We have already mentioned that this important family of febrile diseases, including remittent as well as intermittent fevers, is entirely separated from the genuine Pyrexiæ on the one hand, and from the different species of Miasmatic and Virulent Diseases on the other, and is described as one of the members of the Neuroses. M. B. commences his description of them in these words:

"Like those of a continued type, intermittent and remittent fevers may be either of a miasmatic or of a non-miasmatic origin, *simple* or *compound, i. e.* either consisting of the febrile element alone, or of this element, combined with a septic one proceeding from paludal emanations. The intermittent fevers of the first category are pretty generally *benignant,* whereas those of the second may, if not properly treated from their outset, quickly cause the death of the patient; hence the appellation of *pernicious* has been applied to them."

As we proceed in the narrative, we soon discover that our author's opinions are anything but settled on the ætiological history of intermittent fevers. In some passages, he leaves the impression on the reader's mind that paludal exhalations are the only exciting cause; while, in others, he intimates that a case of genuine ague may be produced by mere alternations of atmospheric temperature. But he will not take upon himself to decide the question; although he does not see in what other manner the not unfrequent occurrence of cases of intermittent fever in Paris is to be accounted for. We should have thought that the exhalations from the filthy Seine, when its waters are low in summer, would have been deemed to have had sufficiently potent influence in this respect.

The proximate cause of intermittent fever is declared to be "an intermittent irritation, a sort of *neuralgia* of the great sympathetic or ganglionary system of nerves, either with or without paludal infection." Its essential character is a simple nervous excitation of the sanguineous system, whereas that of a continued fever is, as we have already seen, a phlegmasia of this system. M. Bouillaud very adroitly lets go the subject of Remittent Fevers; as it is pretty obvious that their phenomena and course do not tally well with the Procrustean limitations assigned to the other two families of febrile diseases. They are therefore quietly allowed to drop out of consideration, the reader hearing little or nothing about them.

The nervous nature of simple ague is very well shewn by the intimate association and dependence of many truly neuralgic affections on malarious influence, and also by the character of the medicines that are known to be most efficacious in relieving both; for example, quinine, arsenic, &c.

It is amusing to observe how anxious M. Bouillaud is, now-a-days, not to be considered a strict disciple of the *physiological school of medicine*, which has hitherto been lauded by him as the depositary of all that was safe and sound in our profession. Treating of the administration of quinine in the treatment of Agues, he points out the necessity of often exhibiting it in full doses, "sans craindre le developpement de cette gastrite, tant redoutée de certains medecins sous le regne de la doctrine dite *physiologique;* mais c'est en pure perte." And to give greater emphasis to this renunciation of out-and-out adherence to the doctrine in question, he tells us in a note that, so far back as 1826, when he published his treatise on so-called Essential Fevers, he did not partake of the *terreur panique* which most of the *pure* disciples of the physiological school entertained on the subject of administering quinine in the early stage of intermittent fever.

He subsequently informs us that he was the first to propose, and employ (?), with a truly surprising success, the use of Digitalis as a succedaneum for quinine in the treatment of simple mild intermittent fever. He was led to this happy therapeutic thought by the theory he had formed as to the seat and nature of the malady. "Considering that it essentially consisted in a sort of periodic irritation of that portion of the nervous system which presides over the phenomena of circulation, and proceeded from the calorification in the substance of all the internal organs, I then drew the conclusion that Digitalis, whose fundamantel property is to diminish the action of that portion of the nervous system, and of the heart and arteries, would be well suited to the treatment of ague." The result of his first experiments exceeded his expectations; but latterly, it would seem, he has recourse upon all occasions to the Quinine. Such is the history of too many of the therapeutic proposals of recent times.

M. Bouillaud seems to be afraid lest some of his brethren might suppose that the cases, in which the Digitalis had effected a cure, were not cases of ague at all; and he therefore takes the trouble to assure us that, before exhibiting the medicine, either he or his head clerk had always witnessed one or more of the attacks of the fever after the patient's admission into the hospital!

Before parting with our author for the present on the subject of Fevers, it may be as well to let our readers know what opinion he has formed upon one of the most important topics connected with the history of this ubiquitous class of diseases—we allude to the influence of what has been called the Medical Constitution of different seasons in modifying their characters, nature, and results. M. Bouillaud seems to consider much that has been written upon this theme as the result rather of meditation in the closet, than of actual experience at the bed-side of the sick. He talks, with an air of almost contemptuous complacency, of anything valuable being discovered at a time when Physics and Chemistry were yet unknown, when Astronomy was not entirely freed from the chimæras of Astrology, and the science of Medicine consisted of little more save the most vague data and most shapeless materials. Such is M. Bouillaud's estimate of the age when Sydenham lived and wrote!

"To form," says he, "an idea of the precarious state of the science upon the point which now occupies us, such as it has been treated of by the ancients, the reader may consult the faithful analysis which I have given of the doctrines of Sydenham, of Stoll, and of Pinel, in my *Clinique Medicale.* I shall content myself with quoting here some passages which refer more especially to our subject.

"According to Sydenham, *continued, epidemic* or *stationary* fevers differ so much in themselves, (toto cœlo), according to the different constitutions of the seasons, that the very same course of treatment, which may have been most successful during the greater part of a year, shall prove useless or positively hurtful towards its decline. Nothing can be plainer or stronger than the assertion of Sydenham; but we shall search in vain for any *positive proofs* of its truth in the work of this writer.

"There is nothing more celebrated than the doctrine of Stoll upon the importance of changing the therapeutic management of diseases, which he nevertheless designates by the same name—that of pleurisy, for instance—according as this or that state of medical constitution prevails.

"We cannot help groaning over the miseries of our nature, when we reflect that those who, under a new kind of superstition, thus believe, without reserve and serious examination, in this sort of *medical astrology*, which they have adorned with the name of the 'doctrine of medical constitutions,' are the very same persons who deny truths of which the most evident and convincing demonstrations are daily given them.

"As for ourselves, who for twenty-five years have passed our life by the bedside of invalids, and have been entrusted with clinical instruction for more than twelve years, we have neglected no means of ascertaining to what point we may believe all that certain practitioners affirm, respecting the influence of the medical constitution upon the effects of remedies in general, and in particular on the treatment by bleeding in the phlegmasiæ. Well, we declare, with the greatest sincerity, and with a mind free from all systematic prejudice, that these assertions rest upon no solid grounds, that they are for the most part in direct opposition to sound experience (an experience which must not be confounded with that which, till lately, we have been contented with), and that the different phlegmasiæ, when treated according to our formula, all the other conditions being at the same time alike, have been uniformly cured with the same success, however different may have been, according to some practitioners, the medical constitutions which have prevailed during this tolerably long space of time."

Very different is the experience of all our best physicians from that of M. Bouillaud. At present, we shall only point to what Dr. Wilson has said upon the subject, as quoted at page 89 of the present number of this Review.

We must reserve to a second article our examination of the other contents of this Medical Nosography. The work is a large and bulky one, demanding no little patient fatigue in its critical perusal, with the view of communicating to others its leading principles and features. It would have been much more agreeable to us if, in the exercise of this labour, we could have conscientiously bestowed praise instead of dealing so much in censure. But the duty we owe to the medical public forbad the former, and, in our judgment, imperatively called for the latter; and we have the less hesitated to express our disapprobation of M. Bouillaud's pyretological opinions, seeing that the subject involved is one not only of prominent theoretical interest, but of the most immediate and paramount practical importance. More lives are annually sacrificed to the pestilence that is generated in our dense and populous towns, the offspring of filth and want, of sorrow and fatigue, and to that which is exhaled from the marsh, the swamp, or the desert, than to all other maladies put together. How necessary then that medical men should make themselves thoroughly acquainted with everything appertaining to the history of Fever; so that, under the guidance of Divine Providence, they may be blessed to be the means of at least mitigating the severity and limiting the extension of the disease, if they cannot hope either to prevent its outbreak or suddenly to arrest its attacks.

In the following article, we shall examine one of the most interesting questions connected with this truly important subject.

